# Use of beta-blockers in patients with ductal carcinoma in situ and risk of invasive breast cancer recurrence: a Swedish retrospective cohort study

**DOI:** 10.1007/s10549-024-07358-y

**Published:** 2024-05-19

**Authors:** Carina Strell, Daniel Robert Smith, Antonis Valachis, Hellén Woldeyesus, Charlotta Wadsten, Patrick Micke, Irma Fredriksson, Aglaia Schiza

**Affiliations:** 1 Department of Immunology, Genetics, and Pathology, Uppsala University, Dag Hammarskjölds Väg 20, 751 85 Uppsala, Sweden; 2 Department of Clinical Medicine, Centre for Cancer Biomarkers CCBIO, University of Bergen, Bergen, Norway; 3 Clinical Epidemiology and Biostatistics, School of Medical Sciences, Örebro University, Örebro, Sweden; 4 Department of Oncology, Faculty of Medicine and Health, Örebro University Hospital, Örebro University, Örebro, Sweden; 5 Department of Oncology, Uppsala University Hospital, Uppsala, Sweden; 6 Department of Surgical and Perioperative Sciences/Surgery, Umeå University, Umeå, Sweden; 7 Department of Surgery, Sundsvall Hospital, Sundsvall, Sweden; 8 Department of Molecular Medicine and Surgery, Karolinska Institutet, Stockholm, Sweden; 9 Department of Breast, Endocrine Tumors and Sarcoma, Karolinska Comprehensive Cancer Center, Karolinska University Hospital, Stockholm, Sweden

**Keywords:** DCIS, Beta-blockers, Breast cancer recurrence

## Abstract

**Background:**

Retrospective observational studies suggest a potential role of beta-blockers as a protective strategy against progression and metastasis in invasive breast cancer. In this context, we investigated the impact of beta-blocker exposure on risk for progression to invasive breast cancer after diagnosis of ductal cancer in situ (DCIS).

**Methods:**

The retrospective study population included 2535 women diagnosed with pure DCIS between 2006 and2012 in three healthcare regions in SwedenExposure to beta-blocker was quantified using a time-varying percentage of days with medication available. The absolute risk was quantified using cumulative incidence functions and cox models were applied to quantify the association between beta-blocker exposure and time from DCIS diagnosis to invasive breast cancer, accounting for delayed effects, competing risks and pre-specified confounders.

**Results:**

The median follow-up was 8.7 years. One third of the patients in our cohort were exposed to beta-blockers post DCIS diagnosis. During the study period, 48 patients experienced an invasive recurrence, giving a cumulative incidence of invasive breast cancer progression of 1.8% at five years. The cumulative exposure to beta-blocker was associated with a reduced risk in a dose-dependent manner, though the effect was not statistically significant.

**Conclusion:**

Our observational study is suggestive of a protective effect of beta-blockers against invasive breast cancer after primary DCIS diagnosis. These results provide rationales for experimental and clinical follow-up studies in carefully selected DCIS groups.

**Supplementary Information:**

The online version contains supplementary material available at 10.1007/s10549-024-07358-y.

## Introduction

Ductal carcinoma in situ (DCIS) of the breast represents a heterogeneous group of precancerous lesions. The incidence of DCIS has dramatically increased due to screening mammography; however, it varies between countries. In Sweden, DCIS comprises approximately 15 percent of all newly screening detected breast cancers [1]. The goal of therapy for DCIS is to prevent the development of invasive breast cancer. The majority of DCIS patients never progress to invasive breast cancer [2, 3]. Nevertheless, due to the uncertainty over which DCIS patients will progress, almost all patients are currently treated by surgery with or without adjuvant radiotherapy, whereas the use of endocrine treatment is still controversial. The consequence is a considerable overtreatment, while at the same time, it is noteworthy that regardless of treatment modalities, a small subgroup of patients will still experience invasive recurrent disease [4]. All treatment modalities are associated with a non-negligible risk of overtreatment and exposure to adverse long-lasting side-effects affecting the patient’s quality of life [5]. Therefore, more effective and less toxic treatment options are highly warranted. The repurposing of drugs, which have proven low toxicity and well tolerability in other medical indications, emerges as an attractive option particularly for this patient group.

Beta-adrenergic receptor antagonists, commonly known as “beta-blockers”, are widely used to treat cardiac and respiratory ailments. Beta-blockers represent a potentially promising group of drugs with a distinctive mode of action, affecting multiple aspects of tumor progression [6]. Moreover, beta-adrenergic receptors are expressed by tumor cells as well as cells of the tumor microenvironment [7], albeit with high intra- and intertumoral heterogeneity [8]. Beta-adrenergic receptors are G-protein coupled receptors and mediate the signaling cascade of catecholamine hormones, which are produced in the context of stress responses. In preclinical models, beta-adrenergic signaling is described to induce the upregulated expression of metastasis-associated genes and downregulate expression of genes facilitating anti-tumor immune responses. These processes could be successfully blocked through administration of non-selective beta-blockers, suggesting a potential pharmacological strategy for prevention of cancer metastases [9–11]. Moreover, some of these studies provided evidence of preclinical activity of beta-blockers in breast cancer models specifically [12–14].

To date, several retrospective studies as well as two meta-analyses concerning the impact of beta-blocker exposure in breast cancer patients have revealed conflicting [9, 15–23] findings. Of note, the majority of these studies included patients with both advanced and early disease stages without specific stratification. However, one recent meta-analysis focusing on early-stage invasive breast cancer noted a potential protective effect of beta-blockers specifically in this patient group [24]. These observations suggest that the efficacy of beta-blockers in preventing breast cancer progression is likely dependent on disease stage and might be most pronounced at early stages. Nevertheless, there is only one randomized clinical trial (phase II) that has investigated the effect of beta blocker usage in breast cancer patients. Results from this study suggest indeed that the use of preoperative propranolol reduced biomarkers of metastatic potential and improved biomarkers of cellular immune response [25]. According to clinicaltrials.gov there are currently two phase II studies in preparation, where propranolol is combined with immunotherapy in advanced or metastatic triple negative breast cancer, but not yet recruiting.

In the light of current evidence, suggesting potential anti-tumor effects of beta-blockers particularly at early breast cancer stages, we hypothesize that exposure to beta-blockers in patients with DCIS is associated with clinical benefits. Hence, the aim of our study was to investigate the potential impact of beta-blocker exposure on risk for invasive breast cancer recurrence in women diagnosed with pure DCIS.

## Patients and methods

### Study population—study design, data sources, participants and data collection

For this register-based retrospective cohort study, all patients with pure DCIS diagnosed between July 1, 2006 and December 31, 2012 were identified through the research database BCBaSe 2.0 [26]. BCBaSe 2.0 was established using data linkage of The Regional Breast Cancer Quality Registries of the Uppsala/Örebro, Stockholm-Gotland and Northern regions of Sweden (1992–2007), National Quality Register for Breast Cancer (NKBC, 2008–2013), the National Patient Register, the Swedish Cancer Register, the Swedish Cause of Death Register, the Swedish Prescribed Drug Register, all held by the National Board of Health and Welfare, as well as the Longitudinal Integration Database for Health Insurance and Labour Market Studies (LISA) and the Total Population Register, managed by Statistics Sweden. Information from these registers is linked using the ten-digit personal identifier numbers assigned to all citizens residing in Sweden. BCBaSe 2.0 has high coverage and data completeness ensuring the validity of data and the generalizability of the study results. Of note, in NKBC only the first cancer for each breast is registered as primary, patients diagnosed with DCIS are reported by NKBC as the primary breast cancer and any subsequent invasive cancer will be registered as a recurrence in the follow-up formula.

The study was performed in line with the principles of the Declaration of Helsinki. Approval of BCBase 2.0 was granted by the Regional Ethics Committee, Stockholm (Approval number: 2013/1272–31/4). All female patients with pure DCIS in BCBaSe 2.0 who underwent definitive surgical treatment without concurrent diagnosis of invasive breast cancer between 2006 and 2012 were included. Men with DCIS, patients with concurrent diagnosis of invasive breast cancer at index date, were excluded. Last day of follow-up was 31 December, 2018. Data on age at diagnosis, histological grade, adjuvant radiotherapy and use of beta-blockers were collected.

### Outcome measures

Our outcome measure was time from diagnosis of pure DCIS to diagnosis of recurrence in the form of invasive breast cancer during the inclusion period, between July 2006 and December 2012, with last day of follow-up 31st December, 2018.

### Exposure to beta-blockers

Beta-blocker use was estimated by record linkage to the Swedish Prescribed Drug Register, which contains information on ATC code, pack defined daily dose (DDD) and date of dispensing. The following ATC codes were used to identify beta-blocker drugs: C07AB02, C07AB07, C07AB03, C07AA05, C07AG02, C07AA07, C07FB02, C07AG01, and C07AA03 (Supplementary Table 1). We calculated the index of exposure to beta-blockers as time-varying, in order to capture differential exposure over follow-up. Knowing exact dispensed dates, as well as corresponding pack DDD, we calculated the sliding-window CMA9 (continuous multiple interval measures of medication availability/gaps) in weekly (7 days) intervals over follow-up [27]. Briefly, the CMA9 is a modification of the medical possession ratio, representing a ratio of days’ supply of beta-blockers. If the exposure could not be estimated in a given window due to no record of prescription, a value of 0 was assigned.

### Statistical analysis

Summary statistics are presented as frequencies and percentages for categorical variables and medians and interquartile ranges for continuous variables.

We computed a cumulative incidence curve for risk of invasive breast cancer in our study population, accounting for competing risks of emigration and death.

We used a series of Cox models to quantify the association of beta blocker exposure on time from diagnosis of DCIS until diagnosis of invasive breast cancer, censoring on date of emigration or death to allow for competing risks. Due to the relatively low number of events (48 occurrences of invasive breast cancer) we carefully selected a limited number of adjustment covariates based on subject matter expertise in order to avoid over-fitting.

In our model the exposure index was fitted as a time-varying cross-basis term to allow for delayed dependencies [28], see Supplementary Methods for further details. We used the following adjustment covariates: radiotherapy (yes, no), age at in situ diagnosis, and the logarithm of diagnosis date fitted as fixed in time adjustment covariates, and stratified on tumor grade (I–II, III, unknown).

The proportional hazards assumption was assessed using unique term and global Chi-squared tests, as well as plots of the Schoenfeld residuals. Effects were presented as Hazard Ratios (HRs) and 95% confidence intervals (CIs); accordingly, we set α = 0.05 for statistical inference.

All statistical analyses were performed using R version 4.3.1 (R Core Team, 2013), relying heavily on the packages AhereR, survival, dlnm, tidycmprsk, gtsummary and the tidyverse suite [29].

## Results

### Characteristics of study cohort

The study population included in total 2535 patients after surgical resection with diagnosed pure DCIS during July 1, 2006 and December 31, 2012 (median age at diagnosis [interquartile range IQR], 59 [50.0, 67.0] years). The median follow-up time was 8.7 years with an IQR of 7.1–10.7 years. Of those, 1134 patients (44.7%) had grade I–II DCIS and 751 (29.6%) had grade III DCIS while grade was unknown for 650 patients (25.6%). Nearly half of the patients (*n* = 1231, 48.6%) received adjuvant radiotherapy. In total, 812 (32.0%) were exposed to any beta-blocker at any time after diagnosis. Patient characteristics are summarized in Table 1.

**Table 1:** DCIS cohort characteristics

Variable	*N* = 2535^a^
Age (years)^b^	59.0 (50.0, 67.0)
Grade	
I–II	1134 (44.7%)
III	751 (29.6%)
Unknown	650 (25.6%)
Radiotherapy	
No	1304 (51.4%)
Yes	1,231 (48.6%)
Exposed to beta blockers^c^	
No	1723 (68.0%)
Yes	812 (32.0%)
Outcome	
Censored	2246 (88.6%)
Invasive	48 (1.9%)
Emigrated	17 (0.7%)
Died	224 (8.8%)
Time in study (years)	8.7 (7.1, 10.7)

^a^Median (IQR); n (%)

^b^Age at breast cancer in situ diagnosis

^c^Exposure anytime over followup

Patients who were exposed to beta-blockers were older and had fewer cases of nuclear grade III DCIS (Table 2).

**Table 2:** Cohort characteristics split by exposure to beta-blockers

Exposure to beta blockers during follow-up		
Variable	No, *N* = 1723^a^	Yes, *N* = 812^a^
Age (years)^b^	56.0 (48.0, 64.0)	64.0 (57.0, 70.0)
Grade		
I-II	763 (44.3%)	371 (45.7%)
III	549 (31.9%)	202 (24.9%)
Unknown	411 (23.9%)	239 (29.4%)
Radiotherapy		
No	878 (51.0%)	426 (52.5%)
Yes	845 (49.0%)	386 (47.5%)
Outcome		
Censored	1,555 (90.2%)	691 (85.1%)
Invasive	39 (2.3%)	9 (1.1%)
Emigrated	15 (0.9%)	2 (0.2%)
Died	114 (6.6%)	110 (13.5%)
Time in study (years)	8.6 (7.0, 10.5)	9.1 (7.2, 10.9)

^a^Median (IQR); n (%)

^b^Age at breast cancer in situ diagnosis

Forty-eight patients progressed to invasive breast cancer during the time of the study. The cumulative incidence of invasive breast cancer progression was 0.16% (95% CI 0.05–0.39%) at one year post diagnosis and increased to 1.8% (95% CI 1.3–2.4%) at five years and 1.9% (95% CI 1.4–2.5%) at 10 years after diagnosis (Supplementary Fig. 1).

### Time varying effect of beta-blocker exposure on progression to invasive breast cancer for patients diagnosed and treated for DCIS

**Fig. 1:**
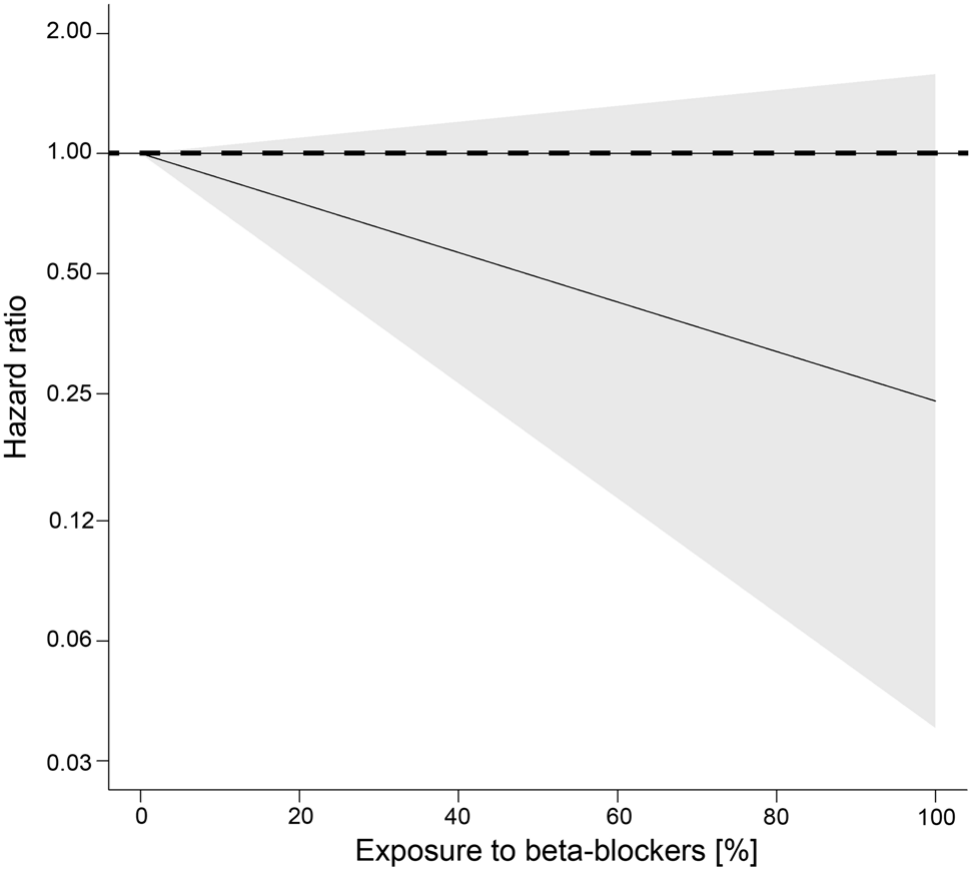
Dose-dependent effect of beta-blocker exposure on progression to invasive breast cancer for women diagnosed and treated for DCIS. Cause-specific cox regression model including exposure as cross-basis term and adjustment for age, grade, radiotherapy and date of DCIS diagnosis. No exposure was set as reference (dashed line). Grey shading indicates the 95% confidence interval

Upon adjustment for age, grade, radiotherapy and date of DCIS diagnosis, exposure to beta-blocker showed a dose-dependent tendency for a decreased risk of invasive breast cancer progression in the cause-specific Cox regression model including exposure as cross-basis term (Fig. 1); however, the results were statistically not-significant.

Considering covariate adjustment and cumulative exposure to beta-blockers, the risk of invasive breast cancer, relative to 0% exposure, decreased in a dose-dependent manner (Table 3). Specifically, patients within the first/lowest quartile of beta-blocker exposure had a HR_adjusted_ of 0.70 [95% CI 0.44–1.12], of the second quartile 0.49 [0.19–1.26], of the third quartile 0.34 [0.08–1.41] and of the fourth/highest quartile 0.24 [0.04–1.58]) (Table 3**)**.

**Table 3:** Dose-dependent effect of beta-blocker exposure on the relative progression risk to invasive breast cancer for DCIS. Adjusted, cause-specific Cox regression model including beta-blocker exposure as cross-basis term

Term	HR	95% CI
BB exposure = 25%^a^	0.700	0.437, 1.121
BB exposure = 50%^a^	0.490	0.191, 1.257
BB exposure = 75%^a^	0.342	0.083, 1.41
BB exposure = 100%^a^	0.240	0.036, 1.581
Age at diagnosis (years)	1.021	0.995, 1.048
Log relative date diagnosis	0.723	0.586, 0.893
Radiotherapy = Yes^b^	0.973	0.547, 1.732

HR's and CI's for BB exposure represent cumulative effects across entire lag period (49 weeks) *HR* hazard ratio *CI* confidence interval *BB*  beta blockers

^a^Reference: BB exposure = 0%

^b^Reference: Radiotherapy = No

In terms of classical clinicopathological characteristics, for each additional year of patient age on diagnosis of DCIS, relative risk of invasive breast cancer increased by 2%, though the effect was non-significant. Radiotherapy reduced the risk of invasive breast cancer by 3%, though again the effect was non-significant. We observed a significantly reduced risk with more recent diagnosis time, consistent with advancement in treatment as well as with the fact that invasive recurrences occur rather later up to 20 years after DCIS diagnosis (Table 3).

## Discussion

This large population-based observational study suggests a potential clinical benefit of beta-blocker exposure on invasive progression in DCIS. The dose-dependent tendency emphasizes the importance of considering the duration and intensity of beta-blocker exposure. Our data align with prior experimental and clinical evidence highlighting the anti-tumor effects of beta-blockers, but now for the first time provide support that already the progression of DCIS to invasive breast might be inhibited by beta-blocker usage.

One of our main findings was that the cumulative exposure to beta-blockers did appear to reduce the risk of invasive breast cancer in a dose-dependent manner, though the associations did not reach statistical significance. Further studies are clearly warranted. While the strengths of our study are the large number of included patients and reliable data including detailed prescription information, several limitations should be acknowledged.

First, the low number of events of invasive progression necessarily limits the power of the applied statistical models. The low number of events of invasive progression is intrinsic for DCIS with its generally very favorable prognosis; however, it might also partly be explained by underreporting of recurrences in two of the three healthcare regions with data included in BCBaSe 2.0 [30]. Wadsten et al. reported a 10% 10-year general recurrence rate (invasive and in situ) after DCIS diagnosis in Uppsala-Örebro 1992–2012, and a validation revealed that only 65% had been registered [31]. Thus, an approximately 13% 10-year recurrence rate for DCIS would probably be more accurate, which is in line with previous randomized trials [1]. Because half of recurrences of DCIS are invasive, the estimated number of invasive events in the present study would be 5–6% opposed to the 1.9% that we report. Invasive recurrences after surgery for pure DCIS occur over a longer time period of up to 20 years post diagnosis and treatment, thus the median follow-up of 8.7 years in our study is still relatively short [32]. Of note, while BCBaSe 2.0 covers three regions representing 60% of Sweden, it doesn't include the entire country. However, 60% has a similar distribution of urban and rural areas and similar socioeconomic status compared to rest 40% (https://www.scb.se/en/), implying minimal negative impact on our results with access to data from all Swedish regions.

Second, in consequence of the low number of invasive events, only a small number of adjustment covariates were selected, those deemed to be the most important confounders using subject matter expertise, in order to avoid over-fitting of the statistical model, thereby compromising its generalizability. Some patient- and tumor-related characteristics differed between patients who were exposed to beta-blockers and those who were not. On average, patients exposed to beta-blockers were older with a lower proportion of grade III DCIS. Although we used multivariable adjustment, it is difficult to rule out confounder imbalances or omitted variable bias. Furthermore, our study focused on beta-blocker exposure post DCIS diagnosis without accounting for exposure to beta-blockers prior to DCIS diagnosis. This pre-diagnostic beta-blocker usage might have impacted the general tumor characteristics and aggressiveness. This question would be of interest to address in future follow-up studies. Finally, we were unable to investigate the potential impact per category and type of beta-blockers due to the limited number of events.

Despite these limitations, it should be highlighted that, to the best of our knowledge, this is the first study to investigate beta-blockers usage in DCIS. Our study is hypothesis-generating and the results are in support of clinical evidence on the potential impact of beta-blockers on disease progression in patients with early breast cancer [24], considering that DCIS is classified as stage 0 breast cancer, namely the earliest stage of breast cancer.

In the era of precision medicine, future in depth tissue analyses are necessary to identify the cellular presence of the drug target as a prerequisite of successful pharmacological intervention. To date, tissue-based analyses of beta-adrenergic receptors in breast cancer only analyzed few patient samples, predominantly of triple negative cases, showing that actually only a small subset of tumor cells is positive for beta-adrenergic expression [33]. This observation indicates that the anti-cancer effects of beta-blocker are likely not due to direct blockage of adrenergic receptors on tumor cells but rather to an inhibition of adrenergic signaling within the tumor microenvironment. Furthermore, these controversies highlight the importance of spatial tissue-based analyses as complement to functional preclinical studies using cancer models. In the optimal scenario, a comparison of histopathological features of diagnostic DCIS tissue samples from patients who were taking beta-blockers at time of diagnosis with those who did not, are strongly motivated. Such data could provide tissue-based evidence on the direct impact of beta-blocker on the underlying biology of breast cancer progression. Interestingly, propranolol may have the ability to revert adrenergic stress mediated immunosuppression [34]. Thus, it may be possible that beta-blocker exposure can enhance the abscopal response following radiation. Given the described effects of adrenergic stress on immune response, future efforts should also aim to investigate potential interactions between beta-blocker exposure and adjuvant radiotherapy. We therefore suggest follow-up studies on DCIS, investigating specifically the efficiency of radiotherapy in the context of beta-blocker exposure.

In conclusion, we present the first study to investigate the potential association of exposure to beta-blockers in patients with pure DCIS and risk of progression to invasive breast cancer. Despite the inherent limitations associated with the observational nature of the study, our findings offer actually new insights of potential clinical importance for DCIS with a beta-blocker dose–response relationship concerning risk for invasive breast cancer recurrence. We hope this work motivates follow-up studies in carefully selected DCIS cohorts with long clinical follow-up. Ideally, such studies should be designed to allow the investigation of specific DCIS subgroups such as high-risk DCIS of different molecular subtypes [35] or DCIS with high tumor infiltrating lymphocytes counts [36] as well as the effect of beta-blocker on radiotherapy efficacy [37].

### Supplementary Information

Below is the link to the electronic supplementary material.

Supplementary file1 (DOC 736 KB)

## Data Availability

Data are available from register holders (Statistics Sweden, Swedish National Board of Health and Welfare, the Regional Cancer Center Stockholm Gotland) for researchers with relevant ethical approvals and who meet the criteria for access to confidential data. The data are not publicly available due to restrictions by Swedish and European law, in order to protect patient privacy.

## Abbreviations

CIs: Confidence intervals
DCIS: Ductal carcinoma in situ
DDD: Defined daily dose
HRs: Hazard ratios
NKBC: National quality register for breast cancer
LISA: Longitudinal integration database for health insurance and labour market studies
