# Supplementary material for: Use of beta-blockers in patients with ductal carcinoma in situ and risk of invasive breast cancer recurrence: a Swedish retrospective cohort study
Source: Breast Cancer Res Treat. 2024 May 19;207(2):293–9. doi: 10.1007/s10549-024-07358-y (PMC11297052; doi:10.1007/s10549-024-07358-y)
Supplement: Supplementary file 1 — Supplementary file1 (DOC 736 KB) [file 10549_2024_7358_MOESM1_ESM.doc]

**Supplementary Material for:**

**Use of beta-blockers in patients with ductal carcinoma in situ and risk of invasive breast cancer recurrence: a Swedish retrospective cohort study**

Carina Strell, Daniel Robert Smith, Antonis Valachis, Hellén Woldeyesus, Charlotta Wadsten, Patrick Micke, Irma Fredriksson, Aglaia Schiza

**Content:**

**Supplementary methods:** *Construction of cross basis term for model 3, index 2*

**Supplementary figure 1:** *Cumulative incidence of progression to invasive breast cancer in the whole DCIS study cohort.*

**Supplementary table 1:** *ATC codes that were used to identify beta-blocker drugs in our cohort study.*

**References**

***Supplementary methods : Construction of cross basis term for model 3, index 2***

At each event time, this model considered beta blocker exposure over the previous 49 weeks. Parametrization of the cross-basis term (1) representing time-varying exposure to beta blockers was chosen by selecting among three competing models using Akaike’s Information Criterion (AIC) as a measure of goodness of fit (2). In all of these three models, the exposure dimension was modelled using a linear function with no intercept to prevent the cross-basis matrix becoming rank-deficient. However, the three models differed in the construction of the lag bases. For the first model, the lag dimension was constructed using a single strata term costing one degree of freedom, equivalent to a standard cumulative exposure index. For the second model, the lag basis was constructed using a beta spline with knots located at 16 and 32 weeks. We imposed a left constraint by excluding the intercept, and a right constraint by excluding two columns of the basis matrix; such parameterization constrains the risk towards the null at the start and end of the lag period. The third model was parameterized the same as the second model, but without the right constraint.

***Supplementary figure 1: Cumulative incidence of progression to invasive breast cancer in the whole DCIS study cohort.*** Emigration and death were treated as competing risks. Grey shading indicates the 95% confidence interval. Numbers of individuals at risk as well as event numbers are indicated in the table.

***Supplementary table 1: ATC codes that were used to identify beta-blocker drugs in our cohort study.***

| C07AB02 | metoprolol |
| --- | --- |
| C07AB07 | bisoprolol |
| C07AB03 | atenolol |
| C07AA05 | propanolol |
| C07AG02 | carvedilol |
| C07AA07 | sotalol |
| C07FB02 | metoprolol and felodipine |
| C07AG01 | labetolol |
| C07AA03 | pindolol |

**References:**

1. Gasparrini, A., B. Armstrong, and M.G. Kenward, *Distributed lag non-linear models.* Stat Med, 2010. **29**(21): p. 2224-34.
2. Sutherland, C., et al., *Practical advice on variable selection and reporting using Akaike information criterion.* Proc Biol Sci, 2023. **290**(2007): p. 20231261.
